# METABOLIC DYSFUNCTION-ASSOCIATED STEATOTIC LIVER DISEASE: UPDATE

**DOI:** 10.1590/S0004-2803.24612025-117

**Published:** 2025-09-05

**Authors:** Maria Gabriela Fernandes DEZAN, Claudia Pinto OLIVEIRA, Helma Pinchemel COTRIM

**Affiliations:** 1 Universidade Federal da Bahia, Hospital Universitário Professor Edgard Santos, Serviço de Gastro-Hepatologia, Salvador, BA, Brasil.; 2 Escola Bahiana de Medicina e Saúde Pública, Salvador, BA, Brasil.; 3 Faculdade de Medicina da Universidade de São Paulo, Departamento de Gastroenterologia, São Paulo, SP, Brasil.; 4 Universidade Federal da Bahia, Programa de Pós-Graduação em Medicina e Saúde, Salvador, BA, Brasil.

**Keywords:** Steatotic liver disease, metabolic dysfunction-associated stea­totic liver disease, MASLD, liver fibrosis, metabolic syndrome, genetic polymorphisms, and drug therapy, Doença hepática esteatótica, doença hepática esteatótica associada à disfunção metabólica, MASLD, fibrose hepática, síndrome metabólica, polimorfismos genéticos e terapia medicamentosa

## Abstract

**Background:**

Since Ludwig proposed the term “nonalcoholic steatohepatitis” (NASH) for this liver disease in 1980, there have been many advances in understanding it, including its epidemiology, pathogenesis, diagnostic methods, and treatment.

**Objective:**

This literature review aims to discuss the most relevant aspects of metabolic dysfunction-associated steatotic liver disease (MASLD).

**Methods:**

The review included clinical studies from the following databases: Embase, PubMed, Scopus, Web of Science, Lilacs, Ovid, and Scopus.

**Results:**

MASLD is the most frequent liver disease worldwide, with increasing prevalence and incidence, and can evolve with liver cirrhosis and hepatocellular carcinoma. The diagnosis involves specific diagnostic criteria involving the presence of hepatic steatosis and other metabolic factors. Drug treatment, still in its incipient, involves pioglitazone, glucagon-like peptide-1 (GLP1) agonists, and sodium glucose cotransporter-2 (SGLT2) inhibitors, especially in diabetic patients. More recently, the Food and Drug Administration (FDA) approved Resmetiron for selected cases.

**Conclusion:**

MASLD is extremely common, presents complex pathophysiology, and requires an intensive multidisciplinary approach. It is hoped that future studies will provide effective and accessible pharmacological therapeutic options for the disease. It is necessary to bring the population’s attention to this condition, which can be associated with significant morbidity and mortality.

## HIGHLIGHTS

• Metabolic dysfunction-associated steatotic liver disease (MASLD) is the most common liver disease globally and a major risk factor for cirrhosis and hepatocellular carcinoma, even without cirrhosis. It refers to steatotic liver disease linked to metabolic syndrome.

• Fibrosis assessment is crucial as it impacts disease prognosis and can be done via non-invasive scores, elastography, or liver biopsy in select cases. MASLD is associated with hepatocellular carcinoma in up to 40% of cases, with 35.5% occurring in advanced fibrosis or cirrhosis.

• Treatment includes GLP-1 agonists and SGLT2 inhibitors, primarily for T2DM and obesity. The FDA recently approved Resmeriton for MASLD with fibrosis stages 2 and 3. Bariatric surgery is an option for patients with BMI ≥35 kg/m² but requires caution in those with advanced fibrosis and portal hypertension due to decompensation risks.

## RESUMO

**Contexto:**

Desde que Ludwig propôs o termo “esteato-hepatite não alcoólica” para esta doença hepática em 1980, houve muitos avanços na sua compreensão, incluindo a sua epidemiologia, patogênese, métodos de diagnóstico e tratamento.

**Objetivos:**

Esta revisão de literatura tem como objetivo de discutir os aspectos mais relevantes da doença hepática esteatótica associada à disfunção metabólica (MASLD).

**Métodos:**

A revisão incluiu estudos clínicos das seguintes bases de dados: Embase, PubMed, Scopus, Web of Science, Lilacs, Ovid e Scopus.

**Resultados:**

MASLD é a doença hepática mais frequente no mundo, com prevalência e incidência crescentes, e que pode evoluir para cirrose hepática e complicar com carcinoma hepatocelular. O diagnóstico perpassa por critérios diagnósticos específicos envolvendo a presença de esteatose hepática e outros fatores metabólicos. O tratamento medicamentoso, ainda incipiente, envolve pioglitazona, agonistas do glucagon-like peptide-1 (GLP1) e inibidores do SGLT2 (*sodium-glucose cotransporter-2*), especialmente para pacientes diabéticos. Mais recentemente, foi aprovado o uso do Resmetiron pelo FDA (*food and drug administration*) para casos selecionados.

**Conclusão:**

A MASLD é extremamente frequente, apresenta fisiopatologia complexa e necessita de abordagem multidisciplinar intensiva. Espera-se que estudos futuros tragam opções terapêuticas farmacológicas efetivas e acessíveis para a doença. É necessário trazer a atenção da população para esta condição que pode se associar a significante morbimortalidade.

## INTRODUCTION

Metabolic dysfunction-associated steatotic liver disease (MASLD) is considered the most common liver disease worldwide and has an estimated prevalence over 30% of the world population^1^. The most frequent risk factors are obesity, type 2 diabetes mellitus (T2DM), and metabolic syndrome. Its broad spectrum includes steatosis, steatohepatitis with or without fibrosis, cirrhosis, and hepatocellular carcinoma. However, it is today recognized as a multisystemic clinical disease, which can be associated with cardiovascular, renal, endocrine diseases, and other clinical conditions^1^ ^-^ ^3^.

This literature review covers MASLD, focusing mainly on changing nomenclature, clinical features, diagnostic criteria, and management. For this purpose, between February 2024 and July 2024, we included clinical studies from the following databases: Embase, PubMed, Scopus, Web of Science, Lilacs, Ovid, and Scopus. The following descriptors along with the Boolean AND and OR systems were used: metabolic-associated steatotic liver disease, liver fibrosis, metabolic syndrome, genetic polymorphisms, and drug therapy. We limited the year of publication to 2010, to aggregate the main developments on the topic over the last 15 years.

## MASLD NOMENCLATURES - NEW DEFINITIONS

In June 2023, the consensus denominated “A multi-society Delphi consensus statement on new fatty liver disease nomenclature” was published introducing a new denomination for nonalcoholic fatty liver disease (NAFLD). The panel chose the term steatotic liver disease (SLD) to encompass the diverse etiologies associated with steatosis. Metabolic-associated steatotic liver disease (MASLD) was chosen as the new term for NAFLD^4^ ^-^ ^5^.

Currently, for MASLD diagnosis, there must be at least one cardiometabolic risk factor in an individual with hepatic steatosis. However, this change in definitions has raised concerns within the scientific community. Could evidences and studies conducted under the old nomenclature NAFLD be extrapolated to people with MASLD? New cohort studies have shown that previous evidence can indeed be applied to individuals with MASLD according to the new definition^1^. Therefore, like the latest EASL-EASD-EASO Clinical Practice Guidelines on this topic, there is evidence on NAFLD that incorporates the most recent findings and uses the terms MASLD and NAFLD synonymously^1^ ^-^ ^6^.

## EPIDEMIOLOGY

The global prevalence of MASLD is estimated over 30%^1^, and several factors influence this rate like genetic background, ethnicity, geographic location, and lifestyle habits such as sedentary lifestyle and dietary patterns^2^ ^-^ ^7^. Over the last 30 years, prevalence in the world has been growing exponentially, rising from rates of 17.6% in the 90s to 23.4% in 2019, showing a growth rate of 1% per year^6^. Regarding incidence, an increase of 60% is estimated between the 1994 - 2006 and 2010 - 2014 periods^6^. A recently published prevalence study evaluated, according to the new definitions, the frequencies of MASLD, metabolic and alcoholic liver disease (MetALD), alcoholic liver disease (ALD), and other etiologies of hepatic steatosis. Steatotic liver disease had a prevalence of 37.8%. In the detailed analysis, MASLD had a prevalence of 32.45% - data similar to those found in previous studies using the previous nomenclature - MetALD 2.56% and ALD 1.17%^8^. Studies carried out in the United States of America (USA) show that Hispanics have a higher prevalence when compared to non-Hispanic white and non-Hispanic black people^7^ ^-^ ^9^. The incidence of MASLD is also on the rise. A cohort study, also carried out in the USA, with an 8-year follow-up, showed that the incidence of MASLD increased by approximately 90%^7^ ^-^ ^9^. In a systematic review with meta-analysis, which included 92 prevalence, 12 incidence, and five mortality studies, Younossi et al.^10^ found that, between 1990 and 2019, the estimated global prevalence of previously called NAFLD was 30.05%. In Latin America, the prevalence has been estimated in 44.37%, followed by the Middle East and North Africa 36.53%, South Asia 33.83%, North America 31.2% East Asia 29.71% Asia-Pacific 28.02% and Western Europe 25.1%.

When evaluating data from non-biopsied patients diagnosed with the disease, the mortality rate per person/year was 12.6 (95%CI: 6.68-23.67) for all causes, 4.2 (95%CI: 1.34-7.05) for cardiac causes, 2.83 (95%CI: 0.78-4.88) for extrahepatic cancers and 0.92 (95%CI: 0-2.21) for liver-related mortality^10^. A 2023 meta-analysis involving more than 100.000 individuals showed that the overall prevalence of previously named NAFLD and NASH in overweight patients was 69.9% and 33.5%, respectively. In patients with obesity, the rates were very similar, 72.3% and 43.1% in patients without and with NASH, respectively^11^. These data show the relevance of this clinical condition not only due to its high prevalence but also due to its association with other morbidities, in addition to the risk of death and cancer.

## RISK FACTORS AND ASSOCIATED DISEASES

The most frequent risk factors are obesity, T2DM, dyslipidemia, and metabolic syndrome. Likewise, systemic arterial hypertension is also commonly associated with MASLD, with its incidence increasing from 6.5 to 14.5 per 100 person-years with the progression of the initial disease to liver cirrhosis. Cardiovascular diseases, which manifest as ischemic heart disease, heart failure, and atrial fibrillation, are complications and relevant causes of death in MASLD^1^ ^,^ ^2^ ^,^ ^7^ ^,^ ^12^.

MASLD can be associated with polycystic ovary syndrome, hypothyroidism, hypogonadism, sarcopenia, and osteoporosis. It is also necessary to investigate obstructive sleep apnea syndrome (OSAS) in these patients, as well as chronic kidney disease, which can influence the progression of liver fibrosis and the prognosis of patients with MASLD^1^ ^,^ ^13^ ^,^ ^14^.

The prevalence of MASLD in individuals with T2DM varies from 30 to 75%, and this is the most significant risk factor in the progression of liver fibrosis and risk of hepatocellular carcinoma (HCC)^2^. Central obesity also has a relevant role in disease progression since body fat distribution is a substantial factor in the association between MASLD and other comorbidities. The android distribution of body fat is related to a greater risk of insulin resistance, cardiovascular disease, and liver fibrosis, regardless of the body mass index (BMI) value^2^.

MASLD individuals also have hormonal changes. Estrogen deficiency in menopausal women may increase the risk of liver fibrosis. In younger women with MASLD, high testosterone levels double the risk of metabolic-associates steato-hepatitis (MASH), liver fibrosis, and abdominal fat deposition. Furthermore, polycystic ovary syndrome is closely linked to MASLD and is independently associated with more severe forms of the disease like steatohepatitis and fibrosis. Finally, subclinical hypothyroidism works as an independent predictor of advanced fibrosis in addition to also predicting the occurrence of steatohepatitis^1^ ^-^ ^3^.

Hepatic and extrahepatic cancers are associated with MASLD due to increased adiposity and a pro-inflammatory state, leading to elevated levels of tumor necrosis factor-α (TNF-α), interleukin-6 (IL-6), and leptin, and reduced adiponectin secretion^12^. This inflammatory environment promotes cell proliferation, tumor growth, angiogenesis, and metastasis. Insulin resistance in these individuals causes hyperinsulinemia and higher levels of insulin-like growth factor-1 (IGF-1), predisposing them to carcinogenesis. Additionally, intestinal dysbiosis linked to MASLD increases intestinal permeability and activates toll-like receptors (TLRs), further raising cancer risk^12^.

## MASLD AND HEPATOCELLULAR CARCINOMA

Among HCC cases worldwide, up to about 40% are associated with MASLD, with approximately 35.5% of cancer cases occurring in patients with advanced fibrosis or liver cirrhosis^15^. The annual incidence of HCC in patients with MASH in Europe and the United States reaches up to 2.6%, and in Brazil, the 5-year incidence reaches around 5%^15^. A cohort study in the USA evaluated 5,098 patients with HCC between 2011 and 2015 and showed that MASLD was the principal etiology found (35.6% with 57.9% cirrhotic individuals). Another relevant finding of this study is that patients with this underlying condition underwent less cancer screening and had lower rates of early detection and a slightly lower survival rate^16^. Although there is still much discussion about the best time to start screening for HCC in patients with MASLD without cirrhosis, current data in the literature recommend that this screening only be carried out in individuals with cirrhosis every 6 months^17^.

## PATHOPHYSIOLOGY

The pathophysiology of MASLD is multifactorial and depends on the genetic background, metabolic factors, insulin resistance, and the intestinal microbiota. 

Several genetic polymorphisms were described, and especially those associated with the polymorphism in the patating-like phospholipase domaincontaining protein 3 (PNPLA3). These polymorphisms have a relevant impact on MASLD susceptibility. PNPLA3 has a connection to the progression of liver disease and the occurrence of HCC in MASH patients with fibrosis and cirrhosis. The I148M variant of PNPLA3 replaces isoleucine with methionine at codon position 148, changing the nucleotide C to G (rs738409 c.444 C>G, p. I148M). The I148M variant of adiponectin (ADPN) has the intracellular function of regulating lipid flow in hepatocytes, belonging to a group of enzymes that metabolize lipids. PNPLA3 has diverse enzymatic functions, including phospholipase activity, triacylglycerol hydrolase activity, and acyl-CoA-dependent or acyl-CoA-independent lipogenic transacetylase activities^18^. Study carried out in Brazil by Mazo, Oliveira et al. demonstrated a prevalence of 47% of the CG genotypes and 21% of the GG genotype, which increases the risk for MASLD by three times compared to the CC genotype^19^. In Latin America, a preliminary study carried out in Chile reported an allele frequency of the high-risk G allele in 59% of the population (the genetic variant is twice as common in Hispanics than African Americans (40% vs 19%)^20^. However, although the G allele worsens the disease, on the other hand, it may be a factor that shows a better response to lifestyle change treatments and bariatric surgery to reduce steatosis^21^.

A better understanding of the intestinal microbiota has also shown its relevance to the progression of MASLD. The intestine-liver axis has aroused interest because bacterial overgrowth secondary to an imbalance in the microbiota can induce damage to the integrity of the intestinal barrier and, through the portal vein, take bacteria and their metabolites such as PAMPs (molecular patterns associated with pathogens) to the liver, causing hepatocellular aggression of MASLD progression^22^.

## CLINICAL ASPECTS AND DIAGNOSIS

To the MASLD diagnosis is important to consider clinical and biochemical aspects, non-invasive serum markers, imaging methods, and liver biopsy, when indicated. MASLD diagnosis requires attention and a multidisciplinary approach^1^ ^,^ ^2^ ^,^ ^23^. Table 1 shows the cardiometabolic risks for MASLD diagnosis. In the presence of one or more of these factors and the absence of significant alcohol consumption (>20 g/day for women and >30 g/day for men), MASLD is diagnosed^1^,^24^,^25^.

**TABLE 1:** Cardiometabolic risks for MASLD diagnosis^1^.

Cardiometabolic risk	Diagnostic criteria
Plasma triglycerides	≥150 mg/dL (or ≥1.7 mmol/L) or lipid-lowering use
HDL-cholesterol	≤39 mg/dL (or ≤1 mmol/L) e ≤50 mg/dL (or ≤1.3 mmol/L) - in men and women, respectively - or lipid-lowering use
Impairment in glucose metabolism	HbA1C 5.7 - 6.4% or FPG 100 - 125 mg/dL or OGTT (2h) 140 - 199 mg/dL → **Prediabetes** or
	HbA1C ≥6.5% or FPG ≥ 126 mg/dL or OGTT (2h) ≥200 mg/dL → **Type 2 diabetes mellitus** or Treatment for type 2 diabetes
Overweight/Obesity	BMI ≥25 kg/m² or ≥23 kg/m² if Asian ethnicity
	Waist circumference
	If European: ≥94 cm (men) and ≥80 cm (women)
	If South Asian and Chinese: ≥90 cm
	(men) and ≥80 cm (women)
	If Japanese: ≥85 cm (men) and ≥90 cm (women)
Blood pressure	≥130 x 85 mmHg or hypertension treatment

HbA1C: glycated hemoglobina; HDL: high density lipoprotein; FPG: fasting plasma glucose; OGTT: oral glucose tolerance test.

Among the non-invasive markers of fibrosis, the most used are the FIB-4 (Fibrosis-4 Index for Liver Fibrosis), and the NAFLD Fibrosis Score (NFS). Other scores that can be used are the APRI (AST to platelet ratio index) and, more recently studied, the ELF (enhanced liver fibrosis) and ADAPT, both based on collagen formation components. More recently, the combination of laboratory and elastography scores has also been proposed, such as MAST (resonance elastography and PDFF + AST), FAST (transient hepatic elastography + AST) and MEFIB (magnetic resonance elastography and FIB-4)^1^.

There are several methods to perform liver elastography for the diagnosis of fibrosis, including transient elastography (TE), point-shear-wave (pSW), 2D-shear-wave (2D-SW), and magnetic resonance (MR) elastography, considering each method’s applicability and limitations. In individuals with MASLD, TE is the most validated method for evaluating liver fibrosis, although MR elastography is the non-invasive method with greater efficacy. Liver stiffness values <8 kilopascals (kPa) on TE exclude advanced fibrosis. When using transient and 2D-SW elastography, stiffness <5 kPa or <9 kPa, respectively, without other clinical signs excludes advanced chronic compensated liver disease^26^ ^,^ ^27^.

**FIGURE 1:**
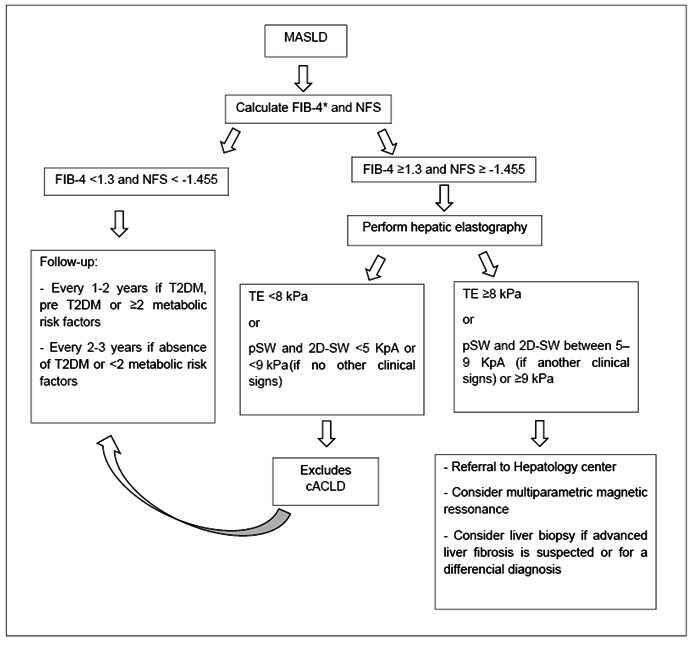
We present the approach to patients with MASLD^1^ ^,^ ^2^ ^,^ ^21^ ^,^ ^23^. Adapted from EASL-EASD-EASO Clinical Practice Guidelines on the management of metabolic dysfunction-associated steatotic liver disease (MASLD) published in 2024^[1]^ and Rinella MA et al. published in 2023^2^.

Patients with non-invasive markers of fibrosis and (or) elastography suggesting advanced fibrosis are candidates for liver biopsy to help in the differential diagnosis with other liver diseases. Histopathological evaluation is still the only method capable of grading necro inflammatory activity and making a more accurate diagnosis of the degrees of fibrosis^2^ ^,^ ^26^. This approach is in Figure 1.

*For >65 years, the FIB-4 cut-off is 2.0. FIB-4 Fibrosis-4 Index for Liver Fibrosis; NFS: NAFLD Fibrosis Score; T2DM: type 2 diabetes mellitus; TE: transient elastography; pSW: point shear-wave; 2D-SW: 2D-shear-wave; cACLD: compensated advanced chronic liver disease.

## TREATMENT

### Lifestyle changes

Lifestyle changes with consequent control of body weight are fundamental measures, especially in people with obesity. Even slight weight loss can have a significant impact, especially on individuals without advanced disease. Losses of 3 to 5% of body weight improve steatosis, and losses >10% can affect steatohepatitis and even fibrosis^2^. For that, patients must be advised to follow a low-calorie diet restricted in refined carbohydrates and saturated fats and encouraged to exercise for at least 150 minutes per week. These orientations must be individualized for each patient, and the participation of nutritionists and physical education professionals is very relevant to adequate results^2^.

### Bariatric surgery and endoscopy

Bariatric surgery is a treatment option for MASLD if BMI ≥35 kg/m². This procedure can reverse MASH and improve the degree of liver fibrosis. However, in patients with cirrhosis and portal hypertension, surgery should be discussed on a case-by-case basis, considering the risks and benefits of the procedure and the Center’s expertise^2^. Another approach that has been emerging in this scenario is endo-bariatric therapies, with an excellent safety and efficacy profile. The principal examples of these procedures are intra-gastric balloon and endoscopic sleeve gastroplasty. Several studies demonstrate that such procedures can improve liver fibrosis seen on histology and elastography and the severity of steatosis. Therefore, multidisciplinary discussion regarding the indication of any of these bariatric procedures for individuals with MASLD is very relevant^28^.

### Drug therapy

The number of clinical trials aiming to evaluate drug therapies for MASLD/MASH has increased exponentially in recent years. Recently, Resmetirom was approved by the FDA as the first drug on the label for MASH with fibrosis 2 and 3. It is a thyroid receptor agonist that has demonstrated histological improvement in MASH and reduction in fibrosis^29^. More effective and safe medications, also approved by the leaflet, are expected soon to treat this condition.

At the moment, some medications can collaborate in the treatment of MASLD, some of them approved for the treatment of comorbidities such as GLP-1 agonists indicated for the treatment of T2DM or obesity, but also with benefit in the MASH treatment^1^ ^,^ ^2^. Phase three studies are underway for MASLD with these drugs. Pioglitazone and vitamin E can be considered with improvement in MASH and stabilization of fibrosis, as suggested by some studies^2^ ^,^ ^30^. Table 2 demonstrates the main therapeutic options available for selected cases of MASLD with their respective benefits and side effects.

**TABLE 2:** Drug therapy with potential benefit on MASLD/MASH^1^ ^,^ ^2^.

Medication	General indication (FDA)	Especial population with liver disease	Clinical effects
**Resmetirom** ^29^	Non-cirrhotic MASH F≥2	Non-cirrhotic MASH F≥2	Benefits: Improve steatohepatitis, fibrosis, glucose and lipidic profiles
			Side effects: gastrointestinal
**Pioglitazone** ^31^	T2DM	MASH with or without T2DM	Benefits: improves MASH, insulin sensitivity, cardiovascular risk reduction
			Side effects: bone loss, weight gain, risk of heart failure exacerbation
**Vitamin E** ^32^	-	MASH without T2DM or cirrhosis	Benefits: improves steatosis
			Side effects: hemorrhagic stroke, prostatic cancer (controversial)
**GLP-1 agonists** ^33^ ^,^ ^34^	T2DM, obesity	MASH without cirrhosis	Benefits: improves steatosis, insulin sensitivity, and cardiovascular outcomes, weigh loss
			Side effects: gastrointestinal (nausea, gastroparesis, diarrhea), gallstones, pancreatitis
**Tirzepatide** ^35^	T2DM	MASLD with T2DM or obesity	Benefits: reduces steatosis, improves insulin sensitivity, weight loss
			Side effects: gastrointestinal, gallstones, pancreatitis
**SGLT2 Inhibitor** ^36^ ^,^ ^37^	T2DM	MASLD with T2DM	Benefits: reduces steatosis, improves cardiovascular and renal outcomes, benefit in heart failure
			Side effects: genitourinary infection, bone loss, volume depletion

T2DM: type 2 diabetes mellitus; SGLT2: sodium glucose cotransporter-2 inhibitor; GLP1: glucagon-like peptide-1. *Adapted from EASL-EASD-EASO Clinical Practice Guidelines on the management of metabolic dysfunction-associated steatotic liver disease (MASLD) ^1^published in 2024 and Rinella MA(2) et al. published in 2023.

## CONCLUSION

This literature review has shown that there are many advances and perspectives in the better understanding and approach to MASLD. This condition presents an elevated global prevalence that requires multidisciplinary diagnostic and therapeutic approaches.

However, advances have been made in the better understanding of its pathogenesis, non-invasive diagnostic methods have progressed, and promising therapeutic prospects with new drugs are expected in the coming years. Public health measures to control obesity and diabetes mellitus are as necessary as effective control for MASLD prevalence and its progression and complications.
